# Treatment patterns and safety outcomes after splenectomy or thrombopoietin receptor agonists as second-line therapy for immune thrombocytopenia: Korean claims-based study

**DOI:** 10.1016/j.rpth.2026.106992

**Published:** 2026-09-29

**Authors:** Hyung-Jun Lim, Kunye Kwak, Changgon Kim, Yoon Seok Choi, Yong Park, Byung Soo Kim, Ki Hoon Ahn, Ka-Won Kang

**Affiliations:** 1 Graduate School of Software and Artificial Intelligence Convergence, Korea University, Seoul, Republic of Korea; 2 Division of Hematology-Oncology, Department of Internal Medicine, Korea University College of Medicine, Seoul, Republic of Korea; 3 Department of Hematology, Catholic Hematology Hospital, Seoul St. Mary’s Hospital, College of Medicine, The Catholic University of Korea, Seoul, Republic of Korea; 4 Department of Obstetrics & Gynecology, Anam Hospital, Korea University College of Medicine, Seoul, Republic of Korea

**Keywords:** immune thrombocytopenia, infections, splenectomy, thromboembolism, thrombopoietin receptor agonist, treatment outcome

## Abstract

**Background:**

Splenectomy and thrombopoietin receptor agonists (TPO-RAs) are recommended second-line therapies for immune thrombocytopenia. Although guidelines recommend tailoring second-line treatments to patient characteristics, this approach is often unclear in real-world practice.

**Objectives:**

We examined claims-based time to next treatment (TTNT) and safety outcomes after splenectomy or TPO-RA as a second-line therapy using nationally representative Korean claims data.

**Methods:**

Patients diagnosed with immune thrombocytopenia between 2002 and 2021 were included. From the eligible cohort, we identified patients whose index second-line treatment was splenectomy (group 1) or TPO-RA without prior splenectomy (group 2). Propensity score matching was performed between patients in both groups who had received corticosteroids as first-line treatment.

**Results:**

After matching, 447 group 1 and 57 group 2 patients were analyzed. Subsequent treatment was administered to 82.3% and 61.4% of patients in groups 1 and 2, respectively. Median TTNTs were 5.80 and 3.03 months in groups 1 and 2, respectively. Estimated TTNT curves diverged after 24 months, with a longer TTNT observed in the TPO-RA group. Pneumococcal infections occurred in 3.1% of patients in group 1 and none in group 2. Venous thromboembolism (VTE) occurred in 10.5% and 15.7% of patients in groups 1 and 2, respectively. The observed time to VTE was shorter in group 2 than in group 1.

**Conclusion:**

Patients treated with TPO-RAs as a second-line therapy showed a longer TTNT after 24 months, whereas VTE occurred earlier in the TPO-RA group. However, these findings should be interpreted with caution because of residual confounding.

## Essentials

• Selecting second-line therapy for immune thrombocytopenia remains clinically challenging.
• Splenectomy was compared with thrombopoietin receptor agonists for time to next treatment and safety.
• Agonists had a longer time to next treatment from 24 months and earlier venous thromboembolism.
• Exploratory findings should be interpreted with caution given unmeasured confounding.

## Graphical abstract

**Figure:**
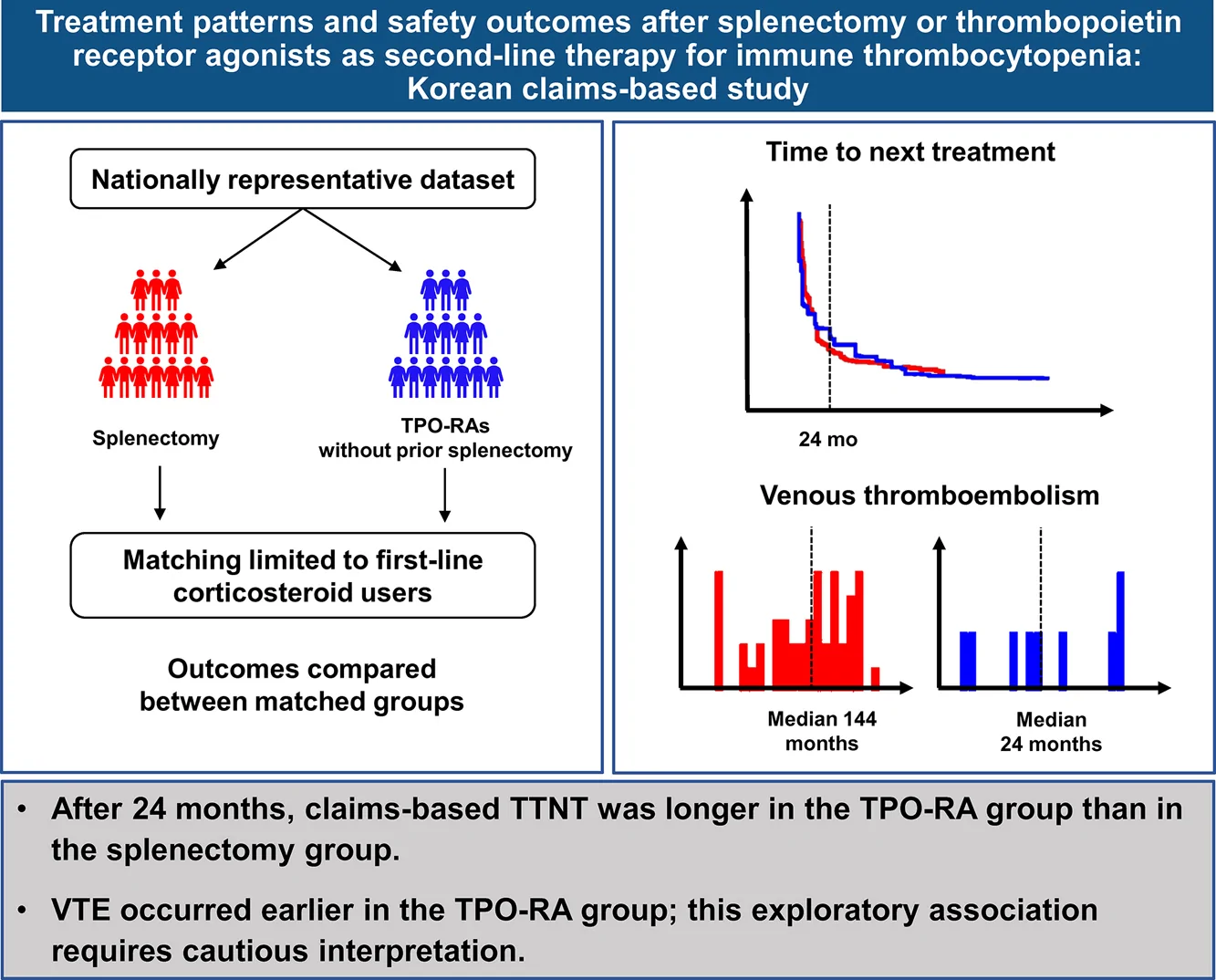

## 1 Introduction

Immune thrombocytopenia (ITP) is an acquired autoimmune bleeding disorder caused by antibody-mediated platelet destruction and impaired platelet production, which presents as isolated thrombocytopenia without other identifiable causes [1,2]. According to the 2019 American Society of Hematology guidelines, corticosteroids are recommended as the first-line treatment for patients with ITP who require treatment [3]. The 2022 American Society of Hematology guideline review reaffirmed this recommendation, emphasizing that corticosteroids remain the cornerstone of ITP treatment and that the current evidence does not warrant a change in the strength or direction of the initial therapy recommendation [4].

For the second-line treatment of ITP, the guidelines recommend either splenectomy or thrombopoietin receptor agonists (TPO-RAs) over rituximab. Although both splenectomy and TPO-RAs are conditionally recommended as second-line options, there is currently no high-certainty evidence directly comparing their efficacy or safety profiles. In the absence of head-to-head randomized trials, the choice between splenectomy and TPO-RAs should be guided by individual patient factors, including comorbidities, treatment goals, lifestyle considerations, and personal preferences [[3], [4], [5]]. Splenectomy is the only treatment capable of inducing long-term, treatment-free remission in a substantial proportion of adult patients with ITP, with sustained response rates of >60% at 5 to 10 years postoperatively [6,7]. However, splenectomy is an invasive and irreversible procedure associated with lifelong risks, including overwhelming postsplenectomy infections and thromboembolic events [8,9]. Although current guidelines recommend the selection of second-line therapies based on individual patient characteristics, these recommendations are unclear in real-world settings. Therefore, more objective and clinically viable information is required to support effective treatment decisions.

Direct comparison of splenectomy and TPO-RAs through randomized clinical trials poses ethical challenges, given that one involves surgery, whereas the other involves pharmacologic therapy. Therefore, we used a nationally representative dataset to examine subsequent treatment patterns and safety outcomes after splenectomy or TPO-RA as a second-line therapy in patients with ITP.

## 2 Materials and Methods

### 2.1 Data source

This study used the National Health Insurance (NHI) claims database, which represents approximately 98% of the population in the Republic of Korea under its universal single-payer health insurance system [10,11]. The NHI collects and anonymizes the billing and treatment data submitted by medical institutions nationwide, enabling its use in population-based research. This study was approved by the Institutional Review Board of Korea University Anam Hospital (Institutional Review Board No. 2025AN0138), and the requirement for informed consent was waived owing to the use of anonymous data. All analyses were performed in compliance with the applicable ethical standards and national research regulations.

### 2.2 Study population

We included individuals from the claims database who met all the following criteria: (1) had a recorded diagnosis of ITP (recorded using the code D693, based on the eighth edition of the Korean Classification of Disease), between January 1, 2002, and December 31, 2021; (2) underwent bone marrow examination within a 3-month window before or after the ITP diagnosis date; and (3) had no record of hematological malignancies, aplastic anemia, or inherited bleeding disorders throughout the observation period. As claims data lack the clinical and laboratory information required to validate primary ITP, a recorded bone marrow examination was included in the claims-based operational definition to improve diagnostic specificity and reduce the potential inclusion of alternative marrow disorders. A ±3-month window was selected to account for possible differences between the timing of clinical diagnostic evaluation and the date of claims-based ITP coding. This criterion was applied consistently to both treatment groups. Detailed diagnostic and procedural codes are provided in Supplementary Table 1. The eighth edition of the Korean Classification of Diseases was announced on July 1, 2020, reflecting the latest updates to the International Classification of Diseases recommended by the World Health Organization and the International Classification of Diseases for Oncology.

### 2.3 Subset of the study population based on second-line therapy

We examined subsequent treatment patterns and safety outcomes after splenectomy or TPO-RA as second-line treatments. To achieve this, a subset of patients was extracted from the overall study population using the following process. First, patients were classified according to their index second-line treatment: splenectomy (group 1) and TPO-RA without prior splenectomy (group 2). Second, within each group, propensity score matching was performed only for patients who received first-line treatment, which was defined as the administration of corticosteroids and/or intravenous immunoglobulin (IVIG) or anti-D immunoglobulins between the date of ITP diagnosis and up to 7 days after splenectomy or initiation of TPO-RA treatment. Splenectomy was recorded using the total splenectomy procedure code (P2091). Treatment with TPO-RAs was defined as the use of eltrombopag (codes 590701ATB and 590702ATB) or romiplostim (codes 615401BIJ and 615402BIJ).

### 2.4 Clinical endpoints

The primary endpoint was claims-based time to next treatment (TTNT), which was identified using an operational definition that we developed for this study. TTNT was defined as the interval from the index treatment to the first subsequent claim for ITP-directed therapy. Under this operational definition, subsequent treatment included corticosteroids, IVIG or anti-D immunoglobulins, TPO-RAs, rituximab, or splenectomy more than 7 days after splenectomy or TPO-RA. A common 7-day grace period was applied to both groups because an early platelet response may occur within the first week after splenectomy and within 1 to 2 weeks after TPO-RA initiation. This was considered the shortest clinically plausible common interval that could be applied to both treatment strategies. Corticosteroids and IVIG or anti-D immunoglobulins were retained in the operational definition because they were principal reimbursed therapies used for additional or rescue treatment of ITP in the Republic of Korea during the study period.

TPO-RA was approved for use in patients with ITP in March 2010 and was covered by the NHI from March 2016 in accordance with the following criteria. It was approved for patients unresponsive to corticosteroids and IVIG, who had either undergone splenectomy or for whom splenectomy was medically contraindicated, with a platelet count of ≤20,000/μL or 20,000 to 30,000/μL, with clinically significant bleeding. If no response was observed after 4 weeks at the maximum dose, then neither continuation nor retreatment was reimbursed. Each course was limited to a maximum of 6 months. Because each reimbursed TPO-RA course was limited to a maximum of 6 months, a TPO-RA prescription recorded more than 6 months after the initial prescription was classified as continued or renewed TPO-RA use. Claims data could not distinguish uninterrupted continuation from reinitiation after a treatment interruption or a separate retreatment course. Therefore, such prescriptions were interpreted as indicating a continued or renewed requirement for TPO-RA treatment rather than initiation of a new treatment line.

The secondary endpoint was the safety of splenectomy and TPO-RAs. Safety assessments included the occurrence of serious infections, specifically pneumococcal disease, *Haemophilus influenzae* infections, and *Neisseria meningitidis* infections, and thromboembolic events. The relevant diagnostic codes are summarized in Supplementary Tables 2 to 4.

### 2.5 Confounder control

In the treatment group comparisons, propensity score matching was used to minimize bias problems that might affect the TTNT and safety profiles. Propensity score matching was performed between patients in groups 1 and 2 who had received first-line treatment, which was defined as the administration of corticosteroids and/or IVIG or anti-D immunoglobulins between the date of ITP diagnosis and up to 7 days after splenectomy or initiation of TPO-RA treatment.

Matching was based on age, sex, Charlson comorbidity index (CCI) (Supplementary Table 5) [12], and the interval between the date of ITP diagnosis and splenectomy or initiation of TPO-RA treatment. Propensity score matching was performed using a caliper width of 0.05. Before matching, baseline characteristics were compared, with continuous variables evaluated using Welch’s *t*-test or an independent *t*-test based on means and variance distributions. Postmatching covariate balance was also evaluated using Welch’s *t*-test or an independent *t*-test based on means and variance distributions, standardized mean differences, and the Kolmogorov-Smirnov test, in addition to visual inspection of propensity score distributions.

### 2.6 Statistical analyses

Continuous variables are summarized as medians and ranges (minimum and maximum) and categorical variables as frequencies and percentages. TTNT was estimated using the Kaplan-Meier method, and the survival curves were compared between the groups using the log-rank test. To account for residual imbalance in measured baseline covariates after matching, multivariable Cox proportional hazards regression was used to estimate the association between the treatment group and TTNT, with adjustment for age, sex, CCI, and the interval between ITP diagnosis and index treatment. The results are reported as adjusted hazard ratios with 95% CIs. To examine whether the observed between-group differences were concentrated during early follow-up, landmark analysis was conducted for periods of 0 to 1, 1 to 6, and 6 to 12 months. Permutation-based sensitivity analyses were performed within predefined time windows of 0 to 1, 1 to 6, 6 to 12, 12 to 24, and 24 to 83 months to evaluate the consistency of the observed between-group differences over time. All statistical analyses were performed using Python (version 3.5.2; Python Software Foundation).

## 3 Results

### 3.1 Baseline characteristics before propensity score matching

A total of 777 patients underwent splenectomy (group 1), and 100 patients received TPO-RA without undergoing splenectomy (group 2) (Table 1). On average, patients in group 2 were older than those in group 1 (median age, 61 and 45 years, respectively; *P* < .001). A significantly higher proportion of patients in group 2 had a CCI of at least 3 than those in group 1 (44% and 27.9%, respectively; *P* < .001). The time from ITP diagnosis to splenectomy was 5.1 (range, 0-213.4) months and that for TPO-RA was 75.5 (range, 0-231.4) months.

**Table 1:** Baseline characteristics before matching.

Baseline characteristics	Total splenectomy (group 1, *n* = 777)	TPO-RA treatment without prior splenectomy (group 2, *n* = 100)	*P*
Age (y), median (range)	45 (0-87)	61 (4-92)	**<.001**
Sex, female, *n* (%)	522 (67.1)	65 (65)	.66
Race and ethnicity	Not available	Not available	—
Charlson comorbidity index, *n* (%)			
0-1	212 (27.2)	27 (27)	**.03**
2	130 (16.7)	12 (12)	.23
≥3	217 (27.9)	44 (44)	**<.001**
Time from ITP diagnosis to total splenectomy or TPO-RA treatment (mo), median (range)	5.1 (0-213.4)	75.5 (0-231.4)	**<.001**
Prior treatment, *n* (%)^a^			
Corticosteroids	447 (57.5)	99 (99)	**<.001**
Intravenous immunoglobulin or anti-D immunoglobulins	343 (44.1)	83 (83)	**<.001**
TPO-RAs	20 (2.5)	100 (100)	.009
Rituximab	0 (0)	1 (1)	—

Values in bold are statistically significant. Race and ethnicity information was not available in the National Health Insurance claims database.

ITP, immune thrombocytopenia; TPO-RA, thrombopoietin receptor agonist.

a “Prior- or post-treatment” was defined based on the treatment initiation date. Treatments administered from the date of ITP diagnosis up to 7 days after splenectomy or the initiation of TPO-RA treatment were classified as prior treatment.

### 3.2 Propensity score matching and baseline characteristics after matching

Propensity score matching was performed between patients in groups 1 and 2 who had received first-line treatment, which was defined as the administration of corticosteroids and/or IVIG or anti-D immunoglobulins between the date of ITP diagnosis and up to 7 days after splenectomy or initiation of TPO-RA treatment. Matching was based on age, sex, CCI, and the interval between the date of ITP diagnosis and splenectomy or initiation of TPO-RA treatment. After matching, the standardized mean difference of the propensity scores was 0.0011, whereas the Kolmogorov-Smirnov statistic was 0.165 (*P* < .001), indicating that distributional differences remained. Although the propensity score distributions showed substantial overlap, clinically important imbalances persisted in several individual covariates (Figure 1).

**Figure 1:**
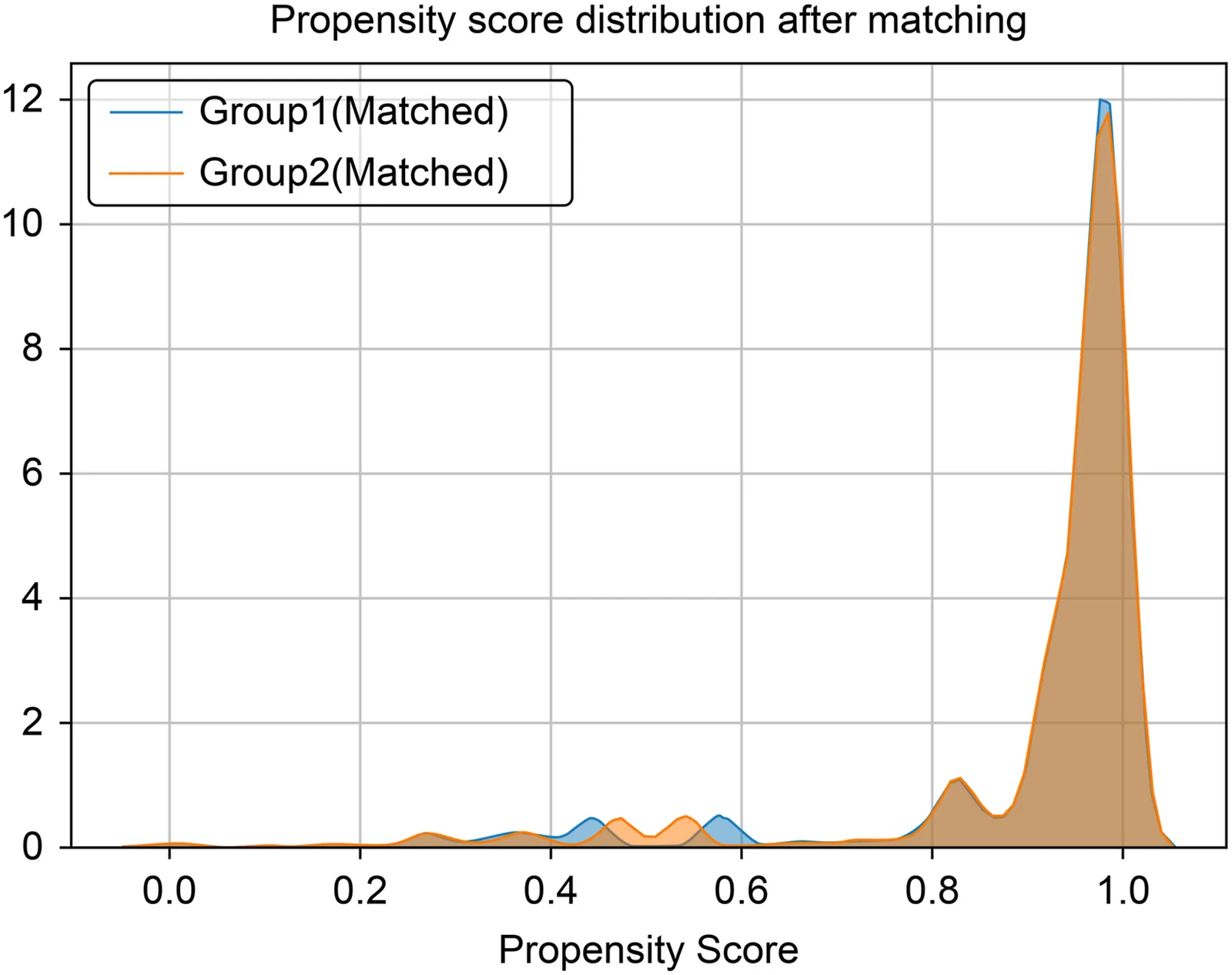
Propensity score distributions. After matching, the propensity score distributions showed substantial overlap. However, the Kolmogorov-Smirnov statistic of 0.165 (*P* < .001) indicated that distributional differences remained.

The baseline characteristics after matching are summarized in Table 2. After matching, 447 and 57 patients in groups 1 and 2, respectively, were included in the final analysis. All patients in both groups were treated with corticosteroids before splenectomy or TPO-RA treatment. In addition, 338 (75.6%) patients in group 1 and 3 (5.2%) patients in group 2 were administered IVIG or anti-D immunoglobulins along with corticosteroids. TTNT and safety outcomes were compared descriptively between the matched groups.

**Table 2:** Baseline characteristics after matching.

Baseline characteristics	Total splenectomy (group 1, *n* = 447)	TPO-RA treatment without prior splenectomy (group 2, *n* = 57)	*P*
Age (y), median (range)	42 (4-87)	64 (4-92)	**<.001**
Sex, female, *n* (%)	311 (69.5)	42 (73.6)	.52
Charlson comorbidity index, *n* (%)			
0-1	133 (29.7)	16 (28)	**.02**
2	75 (16.7)	10 (17.5)	.89
≥3	100 (22.3)	22 (38.5)	**.02**
Time from ITP diagnosis to total splenectomy or TPO-RA treatment (mo), median (range)	5.03 (0-213.4)	27.5 (0-215.1)	**<.001**
Prior treatment, *n* (%)^a^			
Corticosteroids	447 (100)	57 (100)	—
Intravenous immunoglobulin or anti-D immunoglobulins	338 (75.6)	3 (5.2)	**<.001**
TPO-RAs	0 (0)	0 (0)	—
Rituximab	0 (0)	0 (0)	—
Post-treatment, *n* (%)^a^			
Corticosteroids	368 (82.3)	16 (28)	**<.001**
Intravenous immunoglobulin or anti-D immunoglobulins	75 (16.7)	1 (1.7)	**<.001**
TPO-RAs	18 (4)	21 (36.8)	**<.001**
Rituximab	0 (0)	0 (0)	—
Splenectomy	—	0 (0)	—

Values in bold are statistically significant.

ITP, immune thrombocytopenia; TPO-RA, thrombopoietin receptor agonist.

a “Prior- or post-treatment” was defined based on the treatment initiation date. Treatments administered from the date of ITP diagnosis up to 7 days after splenectomy or the initiation of TPO-RA treatment were classified as prior treatment, whereas those provided thereafter were classified as post-treatment. In group 2, a TPO-RA prescription recorded >6 months after initial prescription was classified as continued or renewed TPO-RA utilization.

### 3.3 TTNT after splenectomy or TPO-RA treatment

Subsequent treatment was administered to 368 (82.3%) patients in group 1 and 35 (61.4%) patients in group 2. In group 1, subsequent treatments included corticosteroids (82.3%), IVIG or anti-D immunoglobulins (16.7%), and TPO-RAs (4%). In group 2, the corresponding proportions were 28%, 1.7%, and 36.8%, respectively (Table 2). No patient in group 2 underwent splenectomy after TPO-RA during follow-up. In group 1, most patients received the next treatment within 3 years after splenectomy, whereas in group 2, patients received it within 3 months after the initiation of TPO-RA treatment. The types and frequencies of claims-identified subsequent treatments are shown in Figure 2.

**Figure 2:**
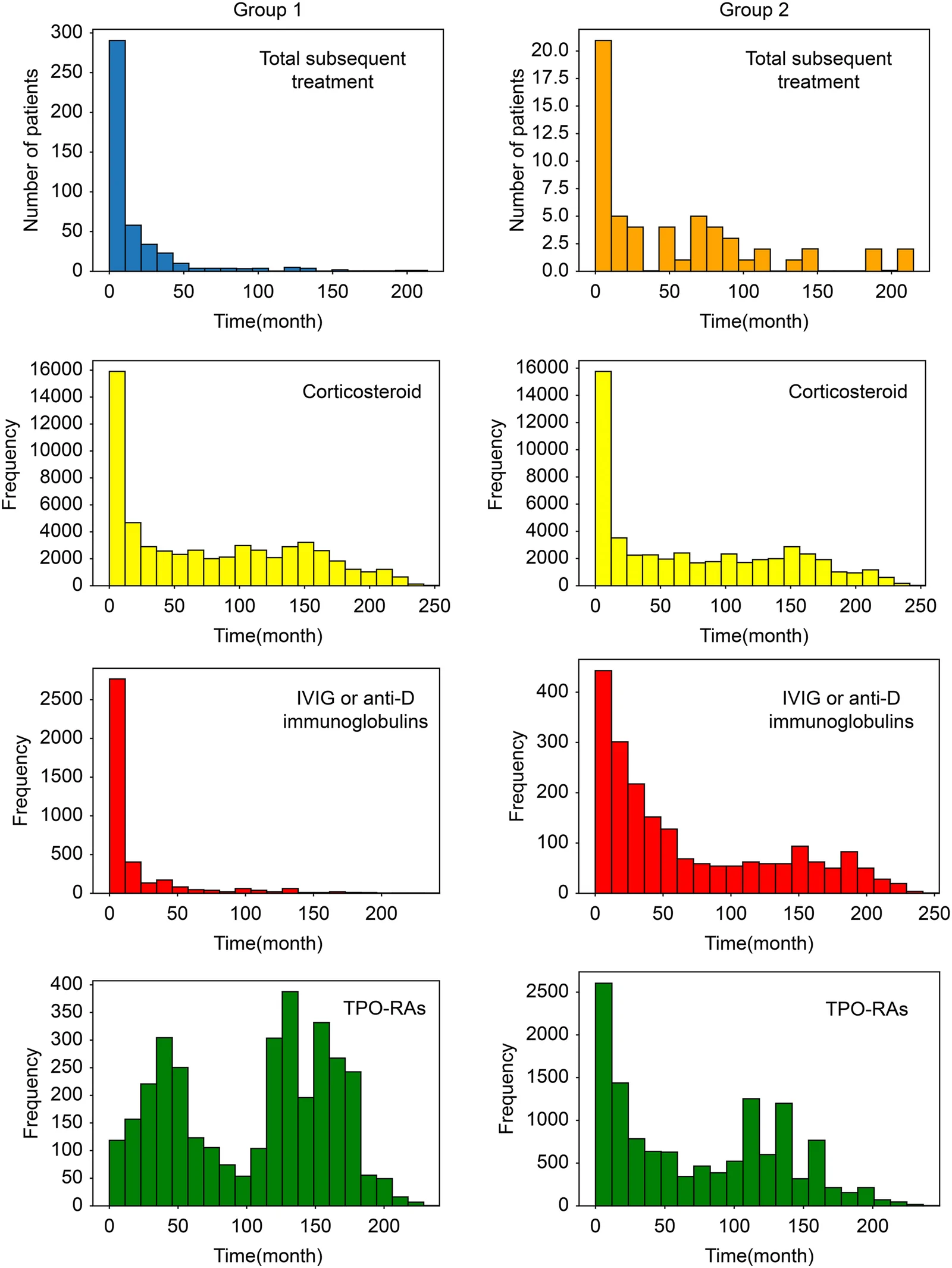
Subsequent treatment patterns in the groups. The frequency was calculated by counting the number of prescription days of the drug for each patient. The cells were counted once per day. IVIG, intravenous immunoglobulin; TPO-RA, thrombopoietin receptor agonist.

The median TTNTs were 5.80 (range, 0-213.46) months in group 1 and 3.03 (range, 0-215.13) months in group 2. During follow-up after splenectomy or initiation of TPO-RA treatment, TTNTs significantly differed between the groups (*P* < .001, Figure 3A). Although propensity score matching was performed, residual imbalances remained: patients in group 2 were older (median, 64 vs 42 years; *P* < .001), more frequently had CCI ≥3 (38.5% vs 22.3%; *P* = .02), and had a longer interval from ITP diagnosis to treatment initiation (median, 27.5 vs 5.03 months; *P* < .001) (Table 2). In the Cox regression analysis, age (hazard ratio [HR], 1.0087; 95% CI, 1.0082-1.009), female sex (HR, 0.6549; 95% CI, 0.6432-0.6677), and CCI (HR, 1.0974; 95% CI, 1.0936-1.1011) were significantly associated with TTNT. The likelihood ratio test was highly significant (χ^2^ = 6515.6, df = 3, *P* < .001). The concordance index was 0.575, indicating limited predictive discrimination, despite the statistical significance of the covariates. The landmark analysis was performed at 0 to 1, 1 to 6, and 6 to 12 months (*P* = .28, <.001, and <.001, respectively). The absence of statistical significance in the earliest interval likely reflects the small sample size and variation in the timing of second-line therapy. Permutation-based sensitivity tests were performed to further evaluate robustness (Figure 3B). In these complementary analyses, a significant difference emerged after 24 months, with a longer estimated TTNT observed in group 2. Before 24 months, these differences were not significant.

**Figure 3:**
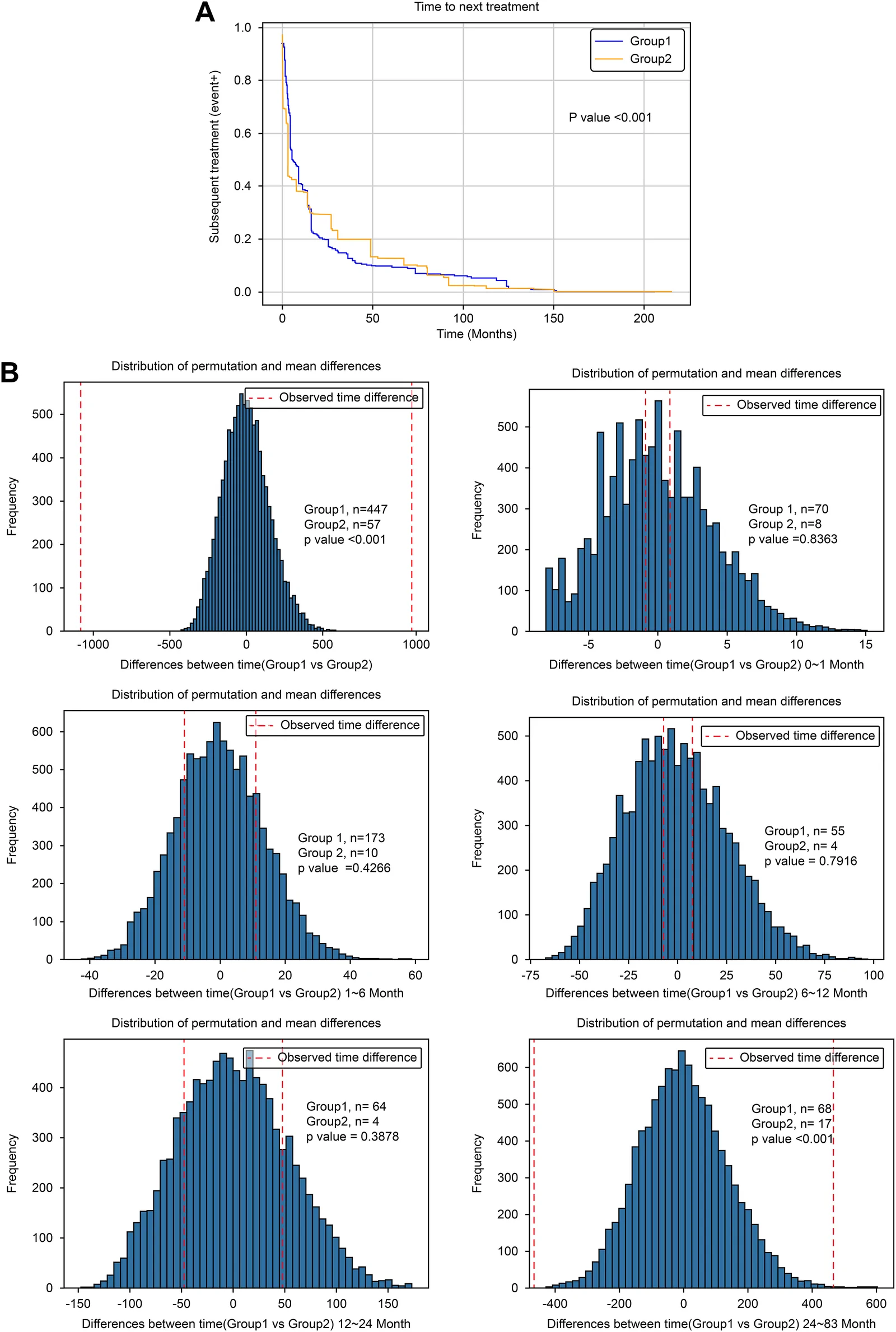
TTNT comparison between groups using sensitivity analysis. (A) TTNT differed significantly between groups during follow-up after splenectomy or TPO-RA treatment initiation (*P* < .001). (B) In permutation-based sensitivity analyses, a significant difference emerged after 24 months, with a longer estimated TTNT in group 2; differences before 24 months were not significant. TPO-RA, thrombopoietin receptor agonist; TTNT, time to next treatment.

### 3.4 Infections after splenectomy or TPO-RA treatment

Pneumococcal infections were reported in 14 (3.1%) patients in group 1 and none in group 2. In group 1, infections occurred at a median of 60.43 (range, 0-182.53) months after treatment. *H. influenzae* infections were reported in 7 (1.5%) patients in group 1 and 1 (1.7%) patient in group 2. In group 1, the infections occurred at a median of 40.13 (range, 20.26-150.03) months after treatment, whereas a single case in group 2 was diagnosed on day 150. *N. meningitidis* infection was not observed in either group.

### 3.5 Thromboembolic events after splenectomy or TPO-RA treatment

Venous thromboembolism (VTE) was observed in 47 of 447 patients (10.5%) in group 1 and 9 of 57 patients (15.7%) in group 2. In group 1, VTE occurred at a median of 144.26 (range, 0-238.53) months after treatment, whereas in group 2, it occurred at a median of 24.3 (range, 3.53-47.16) months (Figure 4A; Supplementary Table 6). Among patients with VTE, the observed median time to VTE was shorter in group 2 than in group 1 (*P* < .001). Arterial thromboembolism (ATE) was observed in 12 (2.6%) patients in group 1 and in 1 (1.7%) patient in group 2. In group 1, ATE occurred at a median of 18.06 (range, 0-179) months after treatment, whereas a single case in group 2 was diagnosed at 1.6 months (Figure 4B; Supplementary Table 6).

**Figure 4:**
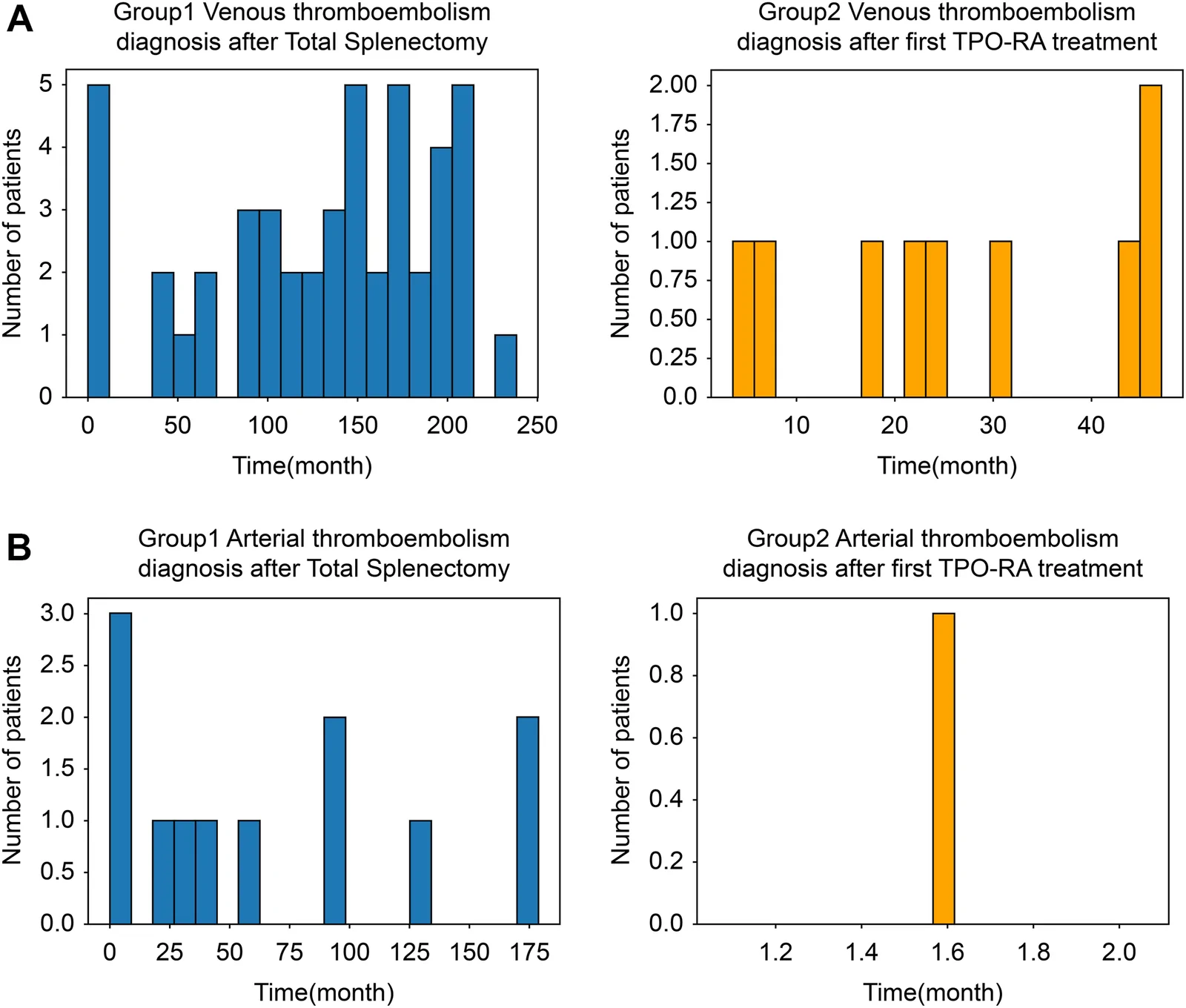
Incidence and timing of thromboembolic events. (A) Among patients with VTE, the observed median time to VTE was shorter in group 2 than in group 1 (24.3 vs 144.3 months; *P* < .001). (B) ATE was observed in 2.6% of group 1 and 1.7% of group 2. ATE, arterial thromboembolism; TPO-RA, thrombopoietin receptor agonist; VTE, venous thromboembolism.

## 4 Discussion

In this real-world analysis, we compared claims-based TTNT and safety outcomes after splenectomy or TPO-RA as a second-line therapy for patients with ITP. A longer TTNT was observed in the TPO-RA group after 24 months. Regarding safety outcomes, pneumococcal infections and ATE were observed in the splenectomy group, whereas VTE occurred earlier in the TPO-RA group.

The NHI claims database that we used covers approximately 98% of the South Korean population, allowing the examination of real-world treatment patterns. Splenectomy was mainly performed in younger patients with lower CCI, and approximately half of the patients underwent the procedure within 6 months of ITP diagnosis (Table 1). In contrast, TPO-RAs without prior splenectomy were administered to older patients with higher CCI and longer intervals between diagnosis and treatment. Most of the 777 splenectomies were performed before 2010 (Supplementary Figure), when TPO-RAs and rituximab were not yet widely available, suggesting that splenectomy was performed relatively early in younger, fit, steroid-refractory, or steroid-dependent patients. For TPO-RAs, the NHI criteria are strict, requiring lack of response to corticosteroids and IVIG, plus either prior splenectomy or medical contraindication, and a platelet count ≤20,000/μL or 20,000 to 30,000/μL, with significant bleeding. Thus, the TPO-RA cohort in this study likely included patients who were medically unfit for splenectomy. According to the Korean ITP Expert Group, such patients include those aged >65 years, with major comorbidities (eg, diabetes, hypertension, cardiac disease, asthma, chronic obstructive pulmonary disease, and immune deficiency), or with factors such as prior extensive abdominal surgery, severe obesity (body mass index >30 kg/m^2^), vaccine allergy, or poor response to IVIG or anti-D immunoglobulin [2]. Therefore, older patients or those with comorbidities are more likely to receive TPO-RAs, and a longer interval from diagnosis to treatment reflects the need to meet these stringent eligibility criteria for insurance coverage.

Clinical studies have reported durable remission after splenectomy and durable platelet responses to TPO-RAs [[13], [14], [15], [16], [17], [18]]. However, these clinically defined response outcomes are not directly comparable to the claims-based TTNT endpoint used in the present study. The higher proportion of patients with claims-identified subsequent treatment may have been primarily attributable to the broad operational definition. As the database does not provide data on platelet counts, bleeding severity, remission status, or the clinical indication for each treatment claim, we could not distinguish continuation or tapering of existing therapy, retreatment, treatment intensification, or temporary rescue treatment. We applied a 7-day grace period to both treatment strategies. However, applying the same grace period does not eliminate potential outcome misclassification because splenectomy and TPO-RAs have different response kinetics and treatment dynamics. Although a longer grace period may have reduced the classification of continued treatment as a subsequent event, it would not reliably distinguish treatment continuation or tapering from retreatment. No significant between-group difference was observed during the first month. However, the landmark and permutation-based analyses did not directly assess or validate alternative grace periods. Accordingly, operationally defined TTNT should be interpreted as a claims-based measure of postindex ITP treatment utilization rather than platelet response, remission, treatment failure, treatment-free survival, or comparative efficacy. In addition, relatively older patients or those with comorbidities were more likely to be included in this study, and their advanced age and underlying conditions may have acted as poor prognostic factors [[19], [20], [21]].

Although most current guidelines place splenectomy and TPO-RAs at the same treatment level, both clinicians and patients clearly prefer to postpone splenectomy, and its use has markedly declined over the past decade, particularly since the approval of TPO-RAs [5]. This trend reflects the availability of alternative second-line options such as TPO-RAs, lack of reliable predictors of response, and potential short- and long-term risks [13,22]. Although most treatments for ITP are more effective when administered earlier, there is no evidence that earlier splenectomy improves outcomes. In addition, a multicenter study reported that patients who received TPO-RAs before splenectomy achieved sustained response rates (approximately 60%) comparable to those observed in the pre–TPO-RA era, and nearly half of those refractory to both TPO-RAs and rituximab still responded to splenectomy [7]. In the present study, a longer estimated TTNT was observed in the TPO-RA group after 24 months. Most patients treated with TPO-RAs required subsequent therapy within 3 months, allowing their response to be evaluated within a relatively short period. Taken together with the results from previous studies, these findings suggest that postponing splenectomy until the response to TPO-RAs has been assessed may be cautiously considered.

To our knowledge, no adequately powered head-to-head studies have compared infection or VTE rates between splenectomy and TPO-RAs in ITP. In this study, the number of infections and thromboembolic events was insufficient to demonstrate a significant difference between the splenectomy and TPO-RA groups. Our interpretation of the infection outcomes was further limited by the absence of information on vaccination status, prophylactic antibiotic use, adherence to postsplenectomy preventive measures, and microbiologic confirmation. VTE occurred earlier among patients in the TPO-RA group than among those in the splenectomy group (median: 24.3 months vs 144.26 months), and 20% of those who developed VTE did so within the first 6 months of TPO-RA treatment. Owing to the limited number of events, persistent differences in age and comorbidity, and unmeasured thrombosis-related factors, the earlier occurrence of VTE cannot be attributed to TPO-RA exposure and should be regarded as an exploratory association. Future studies should aim to verify these results in larger, clinically characterized cohorts.

The primary limitation of this study was that it was based on claims data. Using an operational definition of ITP, we required accurate ITP coding: a bone marrow examination at the index diagnosis; no records of hematological malignancy, aplastic anemia, or inherited bleeding disorders during the observation period; first-line treatment with corticosteroids and/or IVIG or anti-D immunoglobulin; and second-line treatment limited to splenectomy or TPO-RA treatment. Nevertheless, secondary ITP could not be completely excluded and may have been present in the study population. In addition, as mentioned previously, the claims data did not provide information on the degree of bleeding tendency or thrombocytopenia. Therefore, the proposed criteria for assessing the response to ITP treatment cannot be applied. We judged that the definitions of “no response” and “loss of response” in the proposed criteria overlap with the guideline treatment triggers (platelet count ≤30,000/μL or clinically significant bleeding) [2,4,23]. Accordingly, the number of subsequent treatments was counted. However, because the time required to ascertain an adequate response differs between splenectomy and TPO-RAs, an early comparison of sequential treatment-related outcomes between the 2 groups may be inappropriate.

The operational definition of TTNT that we used was derived from the claims data, but it may have introduced bias. The inclusion of corticosteroids, IVIG, and anti-D immunoglobulin may have increased the observed rates of subsequent treatment relative to clinically defined relapse or treatment failure. The common 7-day grace period may have disproportionately affected the TPO-RA group because early dose titration, concomitant therapy, or bridging treatment may extend beyond the first week while awaiting a platelet response. Conversely, extending the grace period could exclude clinically meaningful rescue treatment or evidence of early nonresponse after splenectomy, for which platelet responses frequently occur within the first postoperative week. Thus, neither cutoff would completely eliminate the potential for differential misclassification. In the interval-specific permutation analysis, no significant between-group difference was observed during the first month (*P* = .84), but a significant difference favoring a longer TTNT in the TPO-RA group was observed during the 24- to 83-month interval (*P* < .001). This suggests that treatment claims recorded near the 7-day boundary were unlikely to fully account for the late between-group association. Nevertheless, the sensitivity analyses did not directly validate the 7-day cutoff or exclude treatment misclassification within either group. TTNT should therefore be interpreted as a claims-based measure of postindex treatment utilization rather than clinical relapse or treatment failure. Similarly, TPO-RA prescriptions recorded more than 6 months after initiation could represent uninterrupted continuation, reinitiation after an interruption, or a separate treatment course, which could not be distinguished using claims data.

A methodological limitation of this study was the inadequate balance achieved through propensity score matching, given the limited sample size and substantial baseline covariate imbalances between the groups. Welch’s *t*-test results confirmed that an appropriate balance between the 2 groups was not achieved even after standard propensity score matching. This is a limitation of propensity score matching with small samples with limited common support because restricting the analysis to the region of overlap can reduce the available sample size [24]. In this study, propensity score matching reduced the TPO-RA cohort to 57 patients. To include the remaining 43 patients, who lacked comparable counterparts, would have required extrapolation beyond the observed region of common support and could have exacerbated residual confounding. Therefore, we restricted the primary analysis to the matched cohort to improve comparability with respect to measured baseline covariates while acknowledging the resulting loss of statistical precision and generalizability.

We assessed the consistency of the observed associations through complementary log-rank tests [24], prespecified time-window analyses [25], Cox proportional hazards regression [26], and permutation-based analyses [27]. However, these complementary analyses could not eliminate residual or unmeasured confounding or compensate for limited common support. Accordingly, all between-group differences should be interpreted with caution because clinically important baseline imbalances persisted after matching. As the performance of propensity score matching depends on the selection and specification of measured covariates and cannot account for unmeasured confounding, findings across the entire follow-up period, including those observed beyond 24 months, should be interpreted in this context. The relatively small matched TPO-RA cohort and the limited number of infection and thromboembolic events further reduced statistical precision and increased susceptibility to random error. Although nonparametric methods were used where appropriate, these analyses could not overcome the limited statistical power of the safety comparisons.

The study period ended in 2021 because this was the latest complete year available when the customized NHI dataset was requested and approved. As reimbursed TPO-RA use began only in March 2016, the absence of post-2021 data disproportionately limited both observation period and number of eligible patients in the TPO-RA cohort, thereby further reducing statistical power. Studies using the updated NHI claims database are required to confirm these findings in larger and more contemporary cohorts. Because the NHI is a nationwide administrative claims database established primarily for healthcare administration and reimbursement, self-identified race and ethnicity are not routinely collected. Consequently, we could not evaluate whether treatment patterns and safety outcomes differed across racial or ethnic groups. Although the study population was identified within the Korean national health insurance system, insurance enrollment should not be interpreted as confirmation of Korean ethnicity. This limitation should be considered when generalizing our findings to populations with different racial, ethnic, and healthcare-system characteristics.

Despite having these limitations, a key strength of the study is our use of nationwide real-world data to examine TTNT and safety outcomes after splenectomy or TPO-RA as a secondary therapy. A longer TTNT after 24 months and earlier VTE occurrence were observed in the TPO-RA group. However, these findings are exploratory and should be interpreted cautiously given the potential for residual and unmeasured confounding.

## Figures and Tables

**Figure S1:**
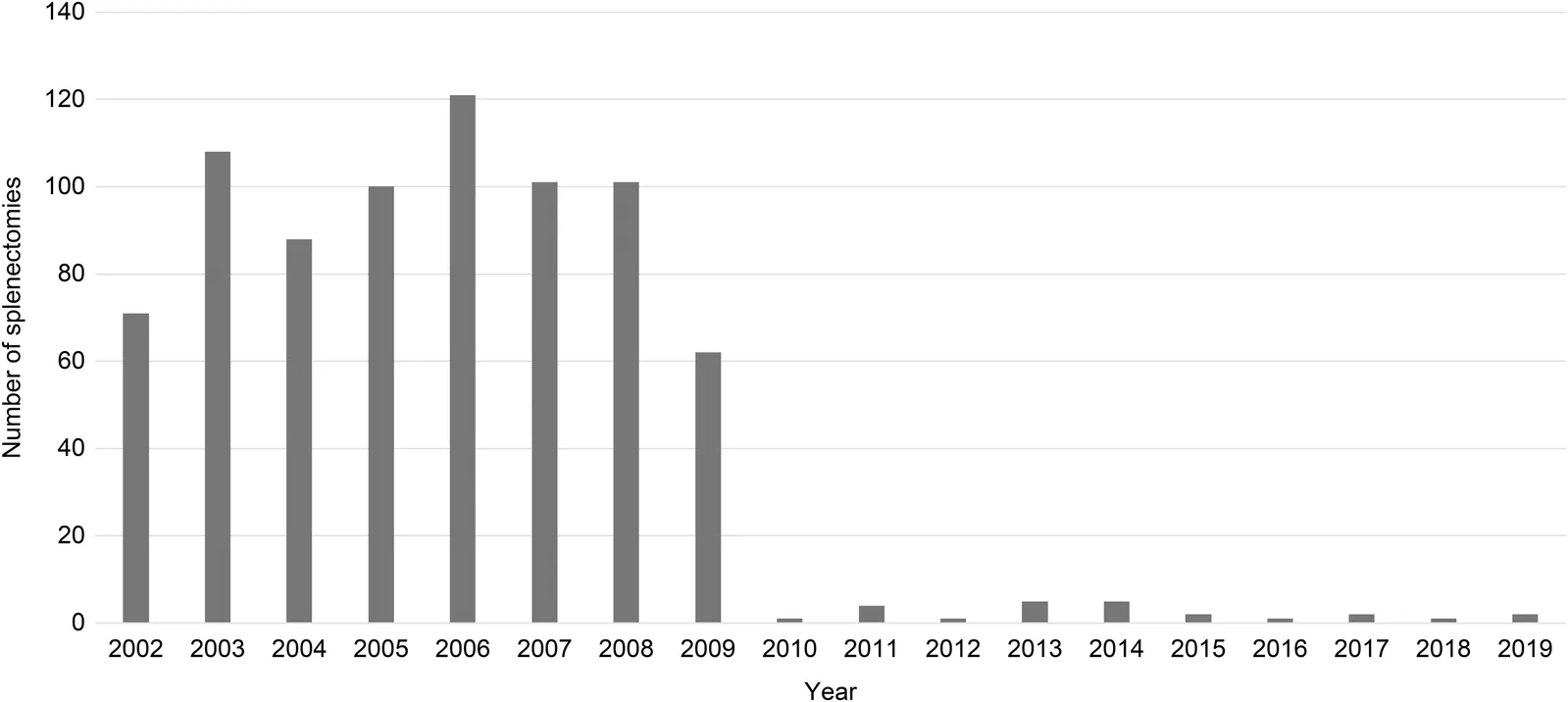


## References

[1] Liu X.-G., Hou Y., Hou M. (2023). How we treat primary immune thrombocytopenia in adults. J Hematol Oncol.

[2] Jang J.H., Kim J.Y., Mun Y.C., Bang S.M., Lim Y.J., Shin D.Y. (2017). Management of immune thrombocytopenia: Korean experts recommendation in 2017. Blood Res.

[3] Neunert C., Terrell D.R., Arnold D.M., Buchanan G., Cines D.B., Cooper N. (2019). American Society of Hematology 2019 guidelines for immune thrombocytopenia. Blood Adv.

[4] Neunert C.E., Arnold D.M., Grace R.F., Kuhne T., McCrae K.R., Terrell D.R. (2024). The 2022 review of the 2019 American Society of Hematology guidelines on immune thrombocytopenia. Blood Adv.

[5] Godeau B. (2023). Is splenectomy a good strategy for refractory immune thrombocytopenia in adults?. Br J Haematol.

[6] Vianelli N., Palandri F., Polverelli N., Stasi R., Joelsson J., Johansson E. (2013). Splenectomy as a curative treatment for immune thrombocytopenia: a retrospective analysis of 233 patients with a minimum follow up of 10 years. Haematologica.

[7] Mageau A., Terriou L., Ebbo M., Souchaud-Debouverie O., Orvain C., Graveleau J. (2022). Splenectomy for primary immune thrombocytopenia revisited in the era of thrombopoietin receptor agonists: new insights for an old treatment. Am J Hematol.

[8] Kristinsson S.Y., Gridley G., Hoover R.N., Check D., Landgren O. (2014). Long-term risks after splenectomy among 8,149 cancer-free American veterans: a cohort study with up to 27 years follow-up. Haematologica.

[9] Provan D., Arnold D.M., Bussel J.B., Chong B.H., Cooper N., Gernsheimer T. (2019). Updated international consensus report on the investigation and management of primary immune thrombocytopenia. Blood Adv.

[10] Kim L., Kim J.-A., Kim S. (2014). A guide for the utilization of health insurance review and assessment service national patient samples. Epidemiol Health.

[11] Cheol Seong S., Kim Y.-Y., Khang Y.-H., Heon Park J., Kang H.J., Lee H. (2017). Data resource profile: the National Health Information database of the National Health Insurance Service in South Korea. Int J Epidemiol.

[12] Charlson M.E., Pompei P., Ales K.L., MacKenzie C.R. (1987). A new method of classifying prognostic comorbidity in longitudinal studies: development and validation. J Chronic Dis.

[13] Chaturvedi S., Arnold D.M., McCrae K.R. (2018). Splenectomy for immune thrombocytopenia: down but not out. Blood.

[14] Kojouri K., Vesely S.K., Terrell D.R., George J.N. (2004). Splenectomy for adult patients with idiopathic thrombocytopenic purpura: a systematic review to assess long-term platelet count responses, prediction of response, and surgical complications. Blood.

[15] Mikhael J., Northridge K., Lindquist K., Kessler C., Deuson R., Danese M. (2009). Short-term and long-term failure of laparoscopic splenectomy in adult immune thrombocytopenic purpura patients: a systematic review. Am J Hematol.

[16] McMillan R., Durette C. (2004). Long-term outcomes in adults with chronic ITP after splenectomy failure. Blood.

[17] Wong R.S.M., Saleh M.N., Khelif A., Salama A., Portella M.S.O., Burgess P. (2017). Safety and efficacy of long-term treatment of chronic/persistent ITP with eltrombopag: final results of the EXTEND study. Blood.

[18] Kuter D.J., Bussel J.B., Lyons R.M., Pullarkat V., Gernsheimer T.B., Senecal F.M. (2008). Efficacy of romiplostim in patients with chronic immune thrombocytopenic purpura: a double-blind randomised controlled trial. Lancet.

[19] Mahévas M., Michel M., Godeau B. (2016). How we manage immune thrombocytopenia in the elderly. Br J Haematol.

[20] Park Y.H., Yi H.G., Kim C.S., Hong J., Park J., Lee J.H. (2016). Clinical outcome and predictive factors in the response to splenectomy in elderly patients with primary immune thrombocytopenia: a multicenter retrospective study. Acta Haematol.

[21] Fabris F., Tassan T., Ramon R., Carraro G., Randi M.L., Luzzatto G. (2001). Age as the major predictive factor of long-term response to splenectomy in immune thrombocytopenic purpura. Br J Haematol.

[22] Wang K.K.W., Charles C., Heddle N.M., Arnold E., Molnar L., Arnold D.M. (2014). Understanding why patients with immune thrombocytopenia are deeply divided on splenectomy. Health Expect.

[23] Rodeghiero F., Stasi R., Gernsheimer T., Michel M., Provan D., Arnold D.M. (2009). Standardization of terminology, definitions and outcome criteria in immune thrombocytopenic purpura of adults and children: report from an international working group. Blood.

[24] Pirracchio R., Resche-Rigon M., Chevret S. (2012). Evaluation of the propensity score methods for estimating marginal odds ratios in case of small sample size. BMC Med Res Methodol.

[25] Gleiss A., Oberbauer R., Heinze G. (2018). An unjustified benefit: immortal time bias in the analysis of time-dependent events. Transpl Int.

[26] Fisher L.D., Lin D.Y. (1999). Time-dependent covariates in the Cox proportional-hazards regression model. Annu Rev Public Health.

[27] Files B.T., Lawhern V.J., Ries A.J., Marathe A.R. (2016). A permutation test for unbalanced paired comparisons of global field power. Brain Topogr.

