# Supplementary material for: Treatment patterns and safety outcomes after splenectomy or thrombopoietin receptor agonists as second-line therapy for immune thrombocytopenia: Korean claims-based study
Source: Res Pract Thromb Haemost. 2026 Sep 29;10(7):106992. doi: 10.1016/j.rpth.2026.106992 (PMC13695308; doi:10.1016/j.rpth.2026.106992)
Supplement: Supplementary file 1 [file mmc1.docx]

**Supplemental material**

**Supplementary Figure 1.** Distribution of splenectomies based on year in group 1

**Supplementary Table 1.** Codes related to study population selection

| **Criterion** | **Code** |
| --- | --- |
| **Included patients** |  |
| Idiopathic thrombocytopenic purpura | D693 |
| **Bone marrow examination (included if any of the following codes were applicable)** |  |
| Bone marrow aspiration smear (with cell percentages) | B1111 |
| Bone marrow aspiration smear (expert reading) | B1113 |
| Bone marrow biopsy | B1115 |
| Bone marrow examination (aspiration, clot section, and biopsy) | B1116 |
| Bone marrow biopsy (expert reading) | B1117 |
| Bone marrow examination (aspiration, clot section, biopsy, and expert reading) | B1118 |
| **Excluded patients (excluded if any of the following codes were applicable)** |  |
| Hematological malignancies |  |
| Hodgkin lymphoma* | C81 |
| Follicular lymphoma* | C82 |
| Non-follicular lymphoma* | C83 |
| Mature T-/NK-cell lymphomas* | C84 |
| Other and unspecified types of non-Hodgkin lymphoma* | C85 |
| Other specified types of T-/NK-cell lymphoma* | C86 |
| Waldenström macroglobulinemia | C880 |
| Extranodal marginal zone B-cell MALT lymphoma* | C884 |
| Multiple myeloma and malignant plasma cell neoplasm* | C90 |
| Lymphoid leukemia* | C91 |
| Myeloid leukemia* | C92 |
| Monocytic leukemia* | C93 |
| Other leukemias of specified cell type* | C94 |
| Leukemia of unspecified cell type* | C95 |
| Other and unspecified malignant neoplasms of lymphoid, hematopoietic, and related tissue* | C96 |
| Other aplastic anemia* | D61 |
| Myelodysplastic syndromes* | D46 |
| Hereditary bleeding disorders |  |
| Hereditary factor Ⅷ deficiency* | D66 |
| Hereditary factor Ⅸ deficiency* | D67 |
| Von Willebrand’s disease | D680 |
| Hereditary factor XI deficiency | D681 |
| Hereditary deficiency of other clotting factors | D682 |
| Qualitative platelet defects (Bernard–Soulier [giant platelet] syndrome, Glanzmann’s disease, gray platelet syndrome, thromboasthenia [hemorrhagic] [hereditary], and thrombocytopathy) | D691 |

Note: Disease classification was performed according to the Korea Classification of Disease (eighth edition) disease classification code. *This entry includes all sub-codes.

Abbreviations: MALT, mucosa-associated lymphoid tissue; NK, natural killer

**Supplementary Table 2.** Codes used to identify ITP treatment

| **Criterion** | **Codes** |
| --- | --- |
| **Corticosteroids** |  |
| Dexamethasone | 141901ATB, 141903ATB, 141904ATB, 142001BIJ, 142030BIJ, 142201BIJ, 142202BIJ, 142230BIJ, 142232BIJ, 142233BIJ |
| Methylprednisolone | 193301ATB, 193302ATB, 193304ATB, 193305ATB, 193501BIJ, 193502BIJ, 193530BIJ, 193531BIJ, 193601BIJ, 193602BIJ, 193603BIJ, 193604BIJ |
| Prednisolone | 217001ATB, 217003ASY, 217004ASY, 217030ASY, 217034ASY, 217035ASY, 217302BIJ |
| **IVIG** | 169902BIJ, 169904BIJ, 169932BIJ, 169935BIJ, 169936BIJ, 169938BIJ, 169940BIJ, 169901BIJ, 169903BIJ, 169905BIJ, 169930BIJ, 169931BIJ, 169933BIJ, 169934BIJ, 169937BIJ, 169939BIJ, 170001BIJ, 170002BIJ, 170003BIJ |
| **Anti-D immunoglobulin** | 173901BIJ, 173902BIJ, 173903BIJ, 173904BIJ, 173930BIJ, 173931BIJ, 173932BIJ |
| **Rituximab** | 422601BIJ, 422602BIJ, 422603BIJ, 422630BIJ, 422631BIJ, 422632BIJ |
| **Splenectomy** | P2091 |
| **TPO-RAs** |  |
| Eltrombopag | 590701ATB, 590702ATB |
| Romiplostim | 615401BIJ, 615402BIJ |

Abbreviations: ITP, immune thrombocytopenia; IVIG, intravenous immunoglobulin; TPO-RA, thrombopoietin receptor agonist

**Supplementary Table 3.** Codes used to identify infections

| **Criterion** | **Codes** |
| --- | --- |
| **Pneumococcal disease** |  |
| Pneumococcal sepsis | A40.3 |
| Pneumococcal meningitis | G00.1 |
| Pneumococcal peritonitis | K67.8 |
| Pneumococcal pneumonia | J13 |
| Pneumococcal infection | A49.1 |
| Pneumococcal endocarditis | I30.1 |
| Pneumococcal arthritis | M00.1* |
| Pneumococcal infections in other sites | B95.3 |
| ***Haemophilus influenzae*** | A41.3, A49.2, G00.0, J14, J20.1, B96.3 |
| ***Neisseria meningitidis*** | A39.0–39.9 |

Note: Disease classification was performed according to the Korea Classification of Disease (eighth edition) disease classification code. *This entry includes all sub-codes.

**Supplementary Table 4.** Codes used to identify thromboembolism

| **Type** | **Criterion** | **Codes** |
| --- | --- | --- |
| **Venous thromboembolism** | **Pulmonary embolism** | I26 |
|  | Pulmonary embolism with mention of acute cor pulmonale | I26.0 |
|  | Pulmonary embolism without mention of acute cor pulmonale | I26.9 |
|  | **Phlebitis and thrombophlebitis** | I80 |
|  | Phlebitis and thrombophlebitis of the superficial vessels of the lower extremities | I80.0 |
|  | Phlebitis and thrombophlebitis of the femoral vein | I80.1 |
|  | Phlebitis and thrombophlebitis of other deep vessels of the lower extremities | I80.2 |
|  | Phlebitis and thrombophlebitis of the lower extremities, unspecified | I80.3 |
|  | Phlebitis and thrombophlebitis of other sites | I80.8 |
|  | Phlebitis and thrombophlebitis of unspecified site | I80.9 |
|  | Portal vein thrombosis | I81 |
|  | **Other venous embolism and thrombosis** | I82 |
|  | Budd–Chiari syndrome | I82.0 |
|  | Thrombophlebitis migrans | I82.1 |
|  | Embolism and thrombosis of the vena cava | I82.2 |
|  | Embolism and thrombosis of the renal vein | I82.3 |
|  | Embolism and thrombosis of the deep veins of the lower extremities | I82.4 |
|  | Embolism and thrombosis of the pelvic veins | I82.5 |
|  | Embolism and thrombosis of other specified deep veins | I82.6 |
|  | Embolism and thrombosis of unspecified deep veins | I82.7 |
|  | Embolism and thrombosis of other specified veins | I82.8 |
|  | Embolism and thrombosis of unspecified vein | I82.9 |
|  | Cerebral infarction due to cerebral venous thrombosis, nonpyogenic | I63.6 |
|  | Nonpyogenic thrombosis of the intracranial venous system | I67.6 |
|  | Superficial thrombosis in pregnancy | O22.0 |
|  | Deep vein thrombosis in pregnancy | O22.1 |
|  | Superficial thrombophlebitis in pregnancy | O22.2 |
|  | Deep phlebothrombosis in pregnancy | O22.3 |
|  | Cerebral venous thrombosis in pregnancy | O22.5 |
|  | Deep phlebothrombosis in the puerperium | O87.1 |
|  | Cerebral venous thrombosis in the puerperium | O87.3 |
|  | Intracranial and intraspinal phlebitis and thrombophlebitis | G08 |
|  | Other diseases of the spinal cord | G95 |
|  | Acute vascular disorders of the intestine | K55.0 |
|  | Chronic vascular disorders of the intestine | K55.1 |
| **Arterial thromboembolism** | Myocardial infarction and other acute ischemic heart disease | I21, I22, I24 |
|  | Cerebral infarction | I63 |
|  | Embolism and thrombosis of the abdominal aorta | I74.0 |
|  | Embolism and thrombosis of other and unspecified parts of the aorta | I74.1 |
|  | Embolism and thrombosis of arteries of the upper extremities | I74.2 |
|  | Embolism and thrombosis of arteries of the lower extremities | I74.3 |
|  | Embolism and thrombosis of the arteries of the extremities, unspecified | I74.4 |
|  | Embolism and thrombosis of the iliac artery | I74.5 |
|  | Embolism and thrombosis of other arteries | I74.8 |
|  | Embolism and thrombosis of the unspecified artery | I74.9 |
|  | Ischemia and infarction of the kidney | N28.0 |

**Supplementary Table 5.** Codes used to identify the Charlson Comorbidity Index

| **Criterion** | **Codes** |
| --- | --- |
| Myocardial infarction | I21, I22, I252 |
| Congestive heart failure | I099, I110, I130, I132, I255, I420, I425, I426, I427, I428, I429, I43, I50, P290 |
| Peripheral vascular disease | I70, I71, I731, I738, I739, I771, I790, I792, K551, K558, K559, Z958, Z959 |
| Cerebrovascular disease | G45, G46, H340, I60, I61, I62, I63, I64, I65, I66, I67, I68, I69 |
| Dementia | F00, F01, F02, F03, F04, F051, G30, G311 |
| Chronic pulmonary disease | I278, I279, J40, J41, J42, J43, J44, J45, J46, J47, J60, J61, J62, J63, J64, J65, J66, J67, J684, J701, J703 |
| Rheumatological disease | M05, M06, M315, M32, M33, M34, M351, M353, M360 |
| Peptic ulcer disease | K25, K26, K27, K28 |
| Mild liver disease | B18, K700, K701, K702, K703, K709, K713, K714, K715, K717, K73, K74, K760, K762, K763, K764, K768, K769, Z944 |
| Diabetes without chronic complication | E100, E101, E106, E108, E109, E110, E111, E116, E118, E119, E120, E121, E126, E128, E129, E130, E131, E136, E138, E139, E140, E141, E146, E148, E149 |
| Diabetes with chronic complication | E102, E103, E104, E105, E107, E112, E113, E114, E115, E117, E122, E123, E124, E125, E127, E132, E133, E134, E135, E137, E142, E143, E144, E145, E147 |
| Hemiplegia or paraplegia | G041, G114, G801, G802, G81, G82, G830, G831, G832, G833, G834, G839 |
| Renal disease | I120, I131, N032, N033, N034, N035, N036, N037, N052, N053, N054, N055, N056, N057, N18, N19, N25, Z490, Z491, Z492, Z940, Z992 |
| Any malignancy, including leukemia and lymphoma | C0, C1, C20, C21, C22, C23, C24, C25, C26, C30, C31, C32, C33, C34, C37, C38, C39, C40, C41, C43, C45, C46, C47, C48, C49, C50, C51, C52, C53, C54, C55, C56, C57, C58, C6, C70, C71, C72, C73, C74, C75, C76, C81, C82, C83, C84, C85, C88, C90, C91, C92, C93, C94, C95, C96, C97 |
| Moderate or severe liver disease | I850, I859, I864, I982, K704, K711, K721, K729, K765, K766, K767 |
| Metastatic solid tumor | C77, C78, C79, C80 |
| Acquired immune deficiency syndrome/human immunodeficiency virus | B20, B21, B22, B24 |

**Supplementary Table 6.** Thromboembolic events after splenectomy or TPO-RA treatment

| **Type** | **Criterion** | **Group 1** | **Group 2** |
| --- | --- | --- | --- |
| **Venous thromboembolism** | **Pulmonary embolism** |  |  |
|  | Pulmonary embolism with mention of acute cor pulmonale |  |  |
|  | Pulmonary embolism without mention of acute cor pulmonale | 9 | 3 |
|  | **Phlebitis and thrombophlebitis** |  |  |
|  | Phlebitis and thrombophlebitis of the superficial vessels of the lower extremities |  |  |
|  | Phlebitis and thrombophlebitis of the femoral vein |  |  |
|  | Phlebitis and thrombophlebitis of other deep vessels of the lower extremities | 12 | 2 |
|  | Phlebitis and thrombophlebitis of the lower extremities, unspecified | 1 | 1 |
|  | Phlebitis and thrombophlebitis of other sites | 3 |  |
|  | Phlebitis and thrombophlebitis of unspecified site | 5 | 1 |
|  | Portal vein thrombosis | 2 |  |
|  | **Other venous embolism and thrombosis** |  |  |
|  | Budd–Chiari syndrome |  |  |
|  | Thrombophlebitis migrans |  |  |
|  | Embolism and thrombosis of the vena cava |  |  |
|  | Embolism and thrombosis of the renal vein |  |  |
|  | Embolism and thrombosis of the deep veins of the lower extremities |  |  |
|  | Embolism and thrombosis of the pelvic veins |  |  |
|  | Embolism and thrombosis of other specified deep veins |  |  |
|  | Embolism and thrombosis of unspecified deep veins |  |  |
|  | Embolism and thrombosis of other specified veins | 2 | 2 |
|  | Embolism and thrombosis of unspecified vein | 11 |  |
|  | Cerebral infarction due to cerebral venous thrombosis, nonpyogenic |  |  |
|  | Nonpyogenic thrombosis of the intracranial venous system |  |  |
|  | Superficial thrombosis in pregnancy |  |  |
|  | Deep vein thrombosis in pregnancy |  |  |
|  | Superficial thrombophlebitis in pregnancy |  |  |
|  | Deep phlebothrombosis in pregnancy |  |  |
|  | Cerebral venous thrombosis in pregnancy |  |  |
|  | Deep phlebothrombosis in the puerperium | 1 |  |
|  | Cerebral venous thrombosis in the puerperium |  |  |
|  | Intracranial and intraspinal phlebitis and thrombophlebitis |  |  |
|  | Other diseases of the spinal cord |  |  |
|  | Acute vascular disorders of the intestine |  |  |
|  | Chronic vascular disorders of the intestine | 1 |  |
| **Arterial thromboembolism** | Myocardial infarction and other acute ischemic heart disease | 1 |  |
|  | Cerebral infarction |  |  |
|  | Embolism and thrombosis of the abdominal aorta |  |  |
|  | Embolism and thrombosis of other and unspecified parts of the aorta |  |  |
|  | Embolism and thrombosis of the arteries of the upper extremities |  |  |
|  | Embolism and thrombosis of the arteries of the lower extremities | 1 |  |
|  | Embolism and thrombosis of the arteries of the extremities, unspecified |  |  |
|  | Embolism and thrombosis of the iliac artery |  |  |
|  | Embolism and thrombosis of other arteries | 5 |  |
|  | Embolism and thrombosis of unspecified artery | 5 | 1 |
|  | Ischemia and infarction of the kidney |  |  |
